# Association of early maladaptive schemas and psychiatric disorders between sex offenders

**DOI:** 10.1192/j.eurpsy.2022.1537

**Published:** 2022-09-01

**Authors:** N. Stepanova, D. Korzun

**Affiliations:** V.P. Serbsky National Medical Research Center of Psychiatry and Narcology, Department Of Psychiatry For Personality Disorder Assistance, Moscow, Russian Federation

**Keywords:** forensic psychiatry, schema-therapy, personality disorder, sex offender

## Abstract

**Introduction:**

Schema-therapy (ST) - one of the promising integrative models of psychotherapy, which shows its efficacy in many mental disorders. ST has main theoretical concepts: early maladaptive schemas (EMS), coping styles, modes and basic needs. EMS are self-defeating emotional and cognitive patterns established from childhood and repeated throughout life. Existing literature shows the connection between EMS and behavioral problems, which could be more significant for patients with personality disorders. The prevalence of personality disorders is relatively low in the general population, but it’s highly overrepresented in the forensic population, especially in groups of sex offenders.

**Objectives:**

The aim of this study is to examine if there is a prevalence of some EMS between sex offenders and their association with a diagnosed psychiatric disorder.

**Methods:**

Medical records and criminal case materials of 27 patients were reviewed, all of them were blamed for committing sex crimes and had to stay at the department for one month for forensic psychiatry examination. During their stay patients were examined several times and questioned with YSQ S3R.

**Results:**

Most of the patients had psychiatric disorder: specific personality disorders (14), pedophilia (3), dependence syndrome (4), organic personality disorder (3). Some of them had several psychiatric diagnoses. The most prevalent EMS were abandonment, emotional deprivation, insufficient self-control and defectiveness.

**Conclusions:**

These findings show the prevalence of personality disorder and several EMS in sex offenders, which could be useful for the full understanding of the concept of PD and improve the organization of medical care for these individuals.

**Disclosure:**

No significant relationships.

